# Application of Circulating Tumor DNA as a Non-Invasive Tool for Monitoring the Progression of Colorectal Cancer

**DOI:** 10.1371/journal.pone.0159708

**Published:** 2016-07-26

**Authors:** Jiaolin Zhou, Lianpeng Chang, Yanfang Guan, Ling Yang, Xuefeng Xia, Liqiang Cui, Xin Yi, Guole Lin

**Affiliations:** 1 Department of General Surgery, Peking Union Medical College Hospital, Chinese Academy of Medical Sciences, Beijing, China; 2 Geneplus-Beijing, Beijing, China; 3 Houston Methodist Research Institute, Houston, Texas, United States of America; Sapporo Medical University, JAPAN

## Abstract

**Background:**

Liquid biopsy has been proposed to be a promising noninvasive tool to obtain information on tumor progression. Through a clinical observation of a case series of 6 consecutive patients, we aim to determine the value of circulating tumor DNA (ctDNA) for monitoring the tumor burden during the treatment of colorectal cancer (CRC).

**Materials and Methods:**

We used capture sequencing of 545 genes to identify somatic alternations in primary tumor tissues of the six CRC patients who underwent radical surgery and in 23 plasma samples collected at serial time points. We compared the mutation patterns and variant allele frequencies (VAFs) between the matched tissue and the plasma samples and evaluated the potential advantage of using ctDNA as a better tumor load indicator to detect disease relapse over carcinoembryonic antigen (CEA), cancer antigen (CA) 19–9 and imaging studies.

**Results:**

We identified low-frequency mutations with a mean VAF of 0.88% (corresponding to a mean tumor burden of 0.20ng/mL) in the preoperative plasmas of four patients with locally advanced CRC and a subset of mutations shared by their primary tumors. The tumor loads appeared a sudden decrease upon surgery or other adjuvant treatments and then generally maintained at low levels (0.092ng/mL) until disease recurred. ctDNA increased by 13-fold when disease relapsed in one patient while the CEA and CA 19–9 levels remained normal. In this patient, all six somatic mutations identified in the preoperative plasma were detected in the recrudescent plasma again, with five mutations showing allele fraction increase.

**Conclusions:**

We described a multi-time-point profile of ctDNA of CRC patients during the course of comprehensive treatment and observed a correlation of ctDNA level with the clinically evaluated tumor progression. This demonstrated a new strategy by analyzing the heterogeneous ctDNA to evaluate and monitor the tumor burden in the treatment and follow-up of CRC patients, with potentially better potency than conventional biomarkers.

## Introduction

With improvements in care in recent decades and the introduction of multidisciplinary treatment, the oncological outcome of colorectal cancer has greatly improved. However, in spite of curatively intended treatment, 30%–40% of these patients will experience recurrence of the disease [1], with postoperative recurrence, and especially widespread metastasis, being the main cause of cancer-related death. According to the literature, about 80% of recurrences after resection of CRC occur within the first two years after surgery [2, 3]. Early disease relapse occurs mainly because the surgical resection was not radical, or unidentified metastasis already existed at the time of surgery. Therefore, the measurement and monitoring of tumor burden is very important during cancer treatment. It informs about radicality of the primary resection and response to adjuvant therapies, and enables proper selection and management of therapeutics and early detection of disease recurrence. Currently, tumor burden is traditionally assessed using circulating biomarkers, including carcinoembryonic antigen (CEA), cancer antigen 19–9 (CA 19–9), and imaging studies, including contrast-enhanced computed tomography (CT) and magnetic resonance imaging (MRI). However, these conventional methods are limited due to their low sensitivity and specificity [4–7]. Consequently, there is a need for better biomarkers for tumor burden measurements.

It has been well recognized that solid tumors, including CRCs, release DNA fragments into the blood, and circulating DNA fragments carrying tumor-specific genetic mutations can be found in patient plasma [8–12]. Recent sequencing studies have shown that virtually all CRCs harbor somatic genetic alterations [13]. Next-generation sequencing (NGS) enables rapid identification of somatic genomic alterations in individual tumors. Further, it enables detection and quantification of these personalized tumor-specific ctDNA fragments in peripheral blood samples, providing a noninvasive method for tumor burden monitoring with very high specificity [9, 14–17].

Until now, ctDNA has not been extensively investigated or compared with other biomarkers of CRC, and it is not clear how relapse affects the ctDNA level. In the present study, we provided a clinical observation to determine the value of ctDNA in the measurement of tumor burden during the course of treatment of CRCs.

## Materials and Methods

### Patients and sample collection

This observational study recruited six patients with primary CRC without distant metastasis who underwent radical resection in our tertiary care center from July 14th, 2011 to August 25th, 2011. Four patients pathologically confirmed as stage III underwent postoperative adjuvant chemotherapy (including one patient with rectal cancer undergoing postoperative concomitant chemoradiotherapy). All patients were regularly followed-up at the out-patient clinic every three months within two years after the surgery and every six months two to five years postoperatively. Tumor tissues were collected during surgery. Blood samples were collected from patients one week before and one month after surgery, and at the routine follow-up visits. The investigators had access to identifying information during and after the data collection. The study was approved by the Ethics Committee of Peking Union Medical College Hospital. All participants provided written informed consent.

### Sample processing and DNA extraction

Tumor DNA was isolated from surgically resected CRC specimens. Matched peripheral blood was collected in EDTA Vacutainer tubes (BD Diagnostics, Franklin Lakes, NJ, USA) and processed within 3 h. Plasma was separated by centrifugation at 2,500 *g* for 10 min, transferred to microcentrifuge tubes, and centrifuged at 16,000 *g* for 10 min to remove remaining cell debris. Peripheral blood lymphocytes (PBLs) from the first centrifugation were used for the extraction of germline genomic DNA. PBL DNA and tumor tissue DNA were extracted using the DNeasy Blood & Tissue Kit (Qiagen, Hilden, Germany). Extracellular (cell-free, cf) circulating DNA was isolated from 0.6–1.8 mL plasma using QIAamp Circulating Nucleic Acid Kit (Qiagen). DNA concentration was measured using a Qubit fluorometer (Invitrogen, Carlsbad, CA USA) and the Qubit dsDNA HS (High Sensitivity) Assay Kit. The size distribution of the cfDNA was assessed using an Agilent 2100 BioAnalyzer and the DNA HS kit (Agilent Technologies, Santa Clara, CA, USA).

### Sequencing library construction and target enrichment

Before library construction, 1 μg each of tissue and PBL DNA was sheared to 300-bp fragments with a Covaris S2 ultrasonicator. Indexed Illumina NGS libraries were prepared from tissue, and PBL germline and circulating DNA libraries were prepared using the KAPA Library Preparation Kit (Kapa Biosystems, Wilmington, MA, USA).

Target enrichment was performed with a custom SeqCap EZ Library (Roche NimbleGen, Madison, WI, USA). To explore the comprehensive genetic properties of CRC, the capture probe was designed based on genomic regions of 545 genes most frequently mutated in CRC and other common solid tumors. Genes and coordinates of selected regions are provided in **Table A in S1 File**. Capture hybridization was carried out according to the manufacturer’s protocol. Following hybrid selection, the captured DNA fragments were amplified and then pooled to generate several multiplex libraries.

### NGS sequencing

Sequencing was carried out using Illumina 2×100 bp paired-end reads on an Illumina HiSeq 2500 instrument according to the manufacturer's recommendations using TruSeq PE Cluster Generation Kit v3 and the TruSeq SBS Kit v3(Illumina, San Diego, CA, USA).

### Sequence data analysis

After removal of terminal adaptor sequences and low-quality data, reads were mapped to the reference human genome and aligned as described previously [18]. GATK (https://www.broadinstitute.org/gatk/, The Genome Analysis Toolkit) and MuTect [19] were employed to call somatic small insertions and deletions (indels) and single nucleotide variants (SNVs) by filtering PBL sequencing data. Contra was used to detect copy number variations [20], and BreakDancer was used to detect cancer-associated structure variations [21]. The final candidate variants were manually verified with the integrative genomics viewer (IGV) brower [22].

### Tumor load analysis

To quantify tumor burden using plasma DNA, nonsynonymous mutations were used as genomic markers. Variant allele frequencies (VAFs) of SNVs and small indels were assessed using the output of SAMtools mpileup. These genomic markers included mutations detected in plasma samples and derived from matched tissue (VAF = 0 when absent in plasma). Absolute ctDNA content in 1 mL plasma was calculated by ctDNA mean fraction multipling cfDNA concentrations.

## Results

### Patient clinical characteristics

All six patients (four females and two males) were diagnosed with primary colorectal carcinoma by pathological examination. Their ages ranged from 57 to 75 years with a median age of 64 years. All patients underwent radical resection of the tumor as the initial treatment. The postoperative histological examinations confirmed R0 resection in all cases and designated one tumor stage I, one tumor stage IIA, and four tumors stage IIIB disease. All patients categorized as stage IIIB received postoperative chemotherapy, including one rectal cancer patient undergoing adjuvant chemoradiotherapy. With a median follow-up of 46 months (range, 32 to 47), one patient died of tumor recurrence with widespread metastasis. Patient characteristics are summarized in **Table B in S1 File**.

### Somatic mutations in tissue

DNA extracted from the surgical tumor tissue was analyzed by capture sequencing to identify somatic genomic alterations. Median sequence coverage of 500× was obtained across all tissue samples, with >99% of the target region covered at ≥20× (**Table C in S1 File**). Overall, the number of nonsynonymous mutations detected in tissues ranged from 6 to 45 (S1 **Fig**), with a mean of 16, including SNVs (89%) and small indels (11%; **Table C in S1 File**). We found that, among the SNVs, C>A/G>T transversions occur most frequently (*N* = 35, 47%), as shown in S2 **Fig**. Besides, no gene included in the target region was identified with significant CNV or other SV in any of samples.

Notably, different driver genes were detected in the six patients, revealing inter-individual tumor genetic heterogeneity (**Table D in S1 File**). For example, TP53 (p.[R175H]) was identified, with VAF of 53% in patient (P)1, whereas KRAS was identified in P3 (p.[G12D]; VAF, ~39%) and P5 (p.[G12V]; VAF, ~35%). Only the APC mutation occurred frequently (in 5/6 patients); most genes were not shared among all patients.

### Somatic mutations in ctDNA

**Fig 1:**
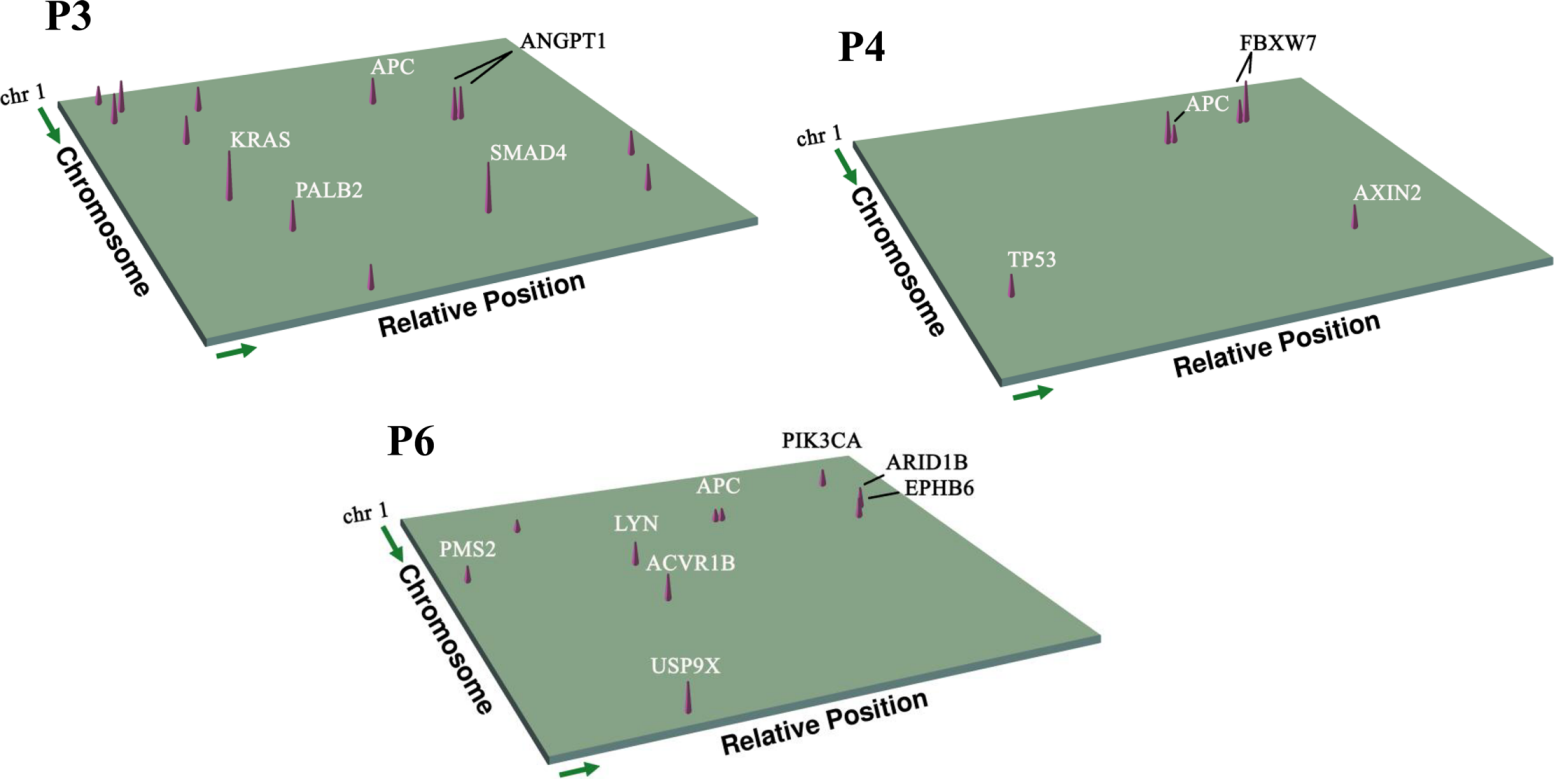
Three-dimensional spectra of genes showing tumor-derived nonsynonymous mutations detected in patient plasma. Spectra are shown for patients 3, 4, and 6. The gene coordinates were plotted according to the reference genome (hg19). Cone height represents relative VAF. Individual driver genes also identified in tissue samples are labeled.

Then, to monitor ctDNA levels in plasma, peripheral blood of six patients was sampled at serial time points (**Table E in S1 File**). Targeted capture sequencing of a total of 23 samples yielded average read depth ranging from 308× to 1436×, with the median capture efficiency of 23% (**Table F in S1 File**). Unfortunately, no mutation was detected in the preoperative plasma of P1 and P2, suggesting the low level of ctDNA in blood of early-stage patients. Of the other preoperative plasma samples, three were observed to share the same mutations with matched tumor tissue. Proportions of these tissue-validated mutations in corresponding preoperative blood results were 50%, 83% and 90%, respectively (**Fig 1, Table G in S1 File**), indicating that ctDNA may contain additional genetic aberrations that had not been captured by the single biopsy tissues obtained at single time points. In the postoperative serial plasma samples, a total of 23 mutations (VAF range, 0.43% to 3.90%) within 19 genes were detected, including 17 SNVs and 6 InDels. Among these SNVs, C>A/G>T transversions (N = 13) showed an overwhelming advantage, which was consistent with the mutation spectrum of the tumor tissues.

### Tumor load

Choosing nonsynonymous somatic SNVs and indels as molecular markers, and clustering of the sets of all tissue-derived mutations and those verified in plasma, the ctDNA fraction at each time point was analyzed based on the average VAF, with mean value of 0.587% in preoperative samples, and 0.133% in postoperative samples. Correspondingly, the mean absolute ctDNA concentration was calculated to be 0.163 and 0.038 ng/mL in pre- and post-surgery samples, respectively (**Table H in S1 File**).

### Postoperative monitoring using ctDNA

To determine whether ctDNA could reflect disease progression, we analyzed tumor burden in serial blood samples of all patients. Tumor burden generally decreased after resection. For patients without relapse, ctDNA was maintained at a stable low level upon intervention with chemotherapy as well as during the follow-up period.

**Fig 2:**
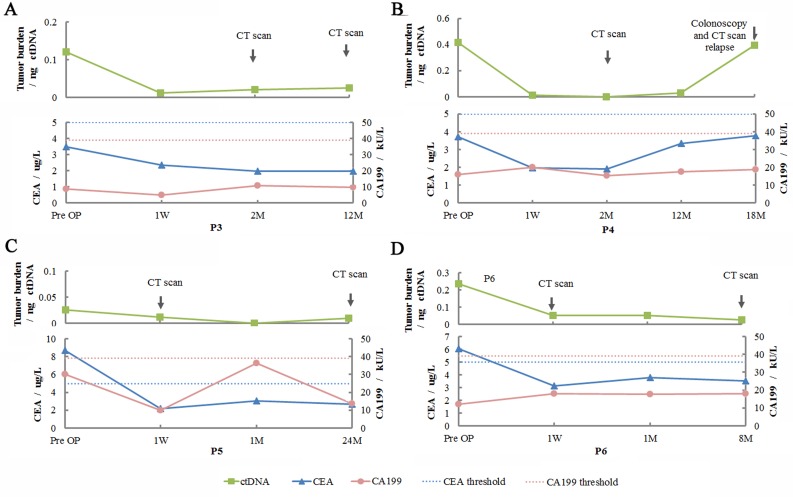
Clinical application of ctDNA to monitor colorectal cancer in patients after surgery. CEA and CA 19–9 levels and tumor burden were assessed at the indicated time points in (**A**) patient 3, (**C**) patient 5, and (**D**) patient 6, who showed no recurrence; (**B**) patient 4, in whom colonoscopy and contrast-enhanced computed tomography (CT) showed recurrence. ctDNA, circulating tumor DNA; P, patient; CEA, carcinoembryonic antigen; CA 19–9, cancer antigen 19–9; W, week; M, month(s).

**Fig 3:**
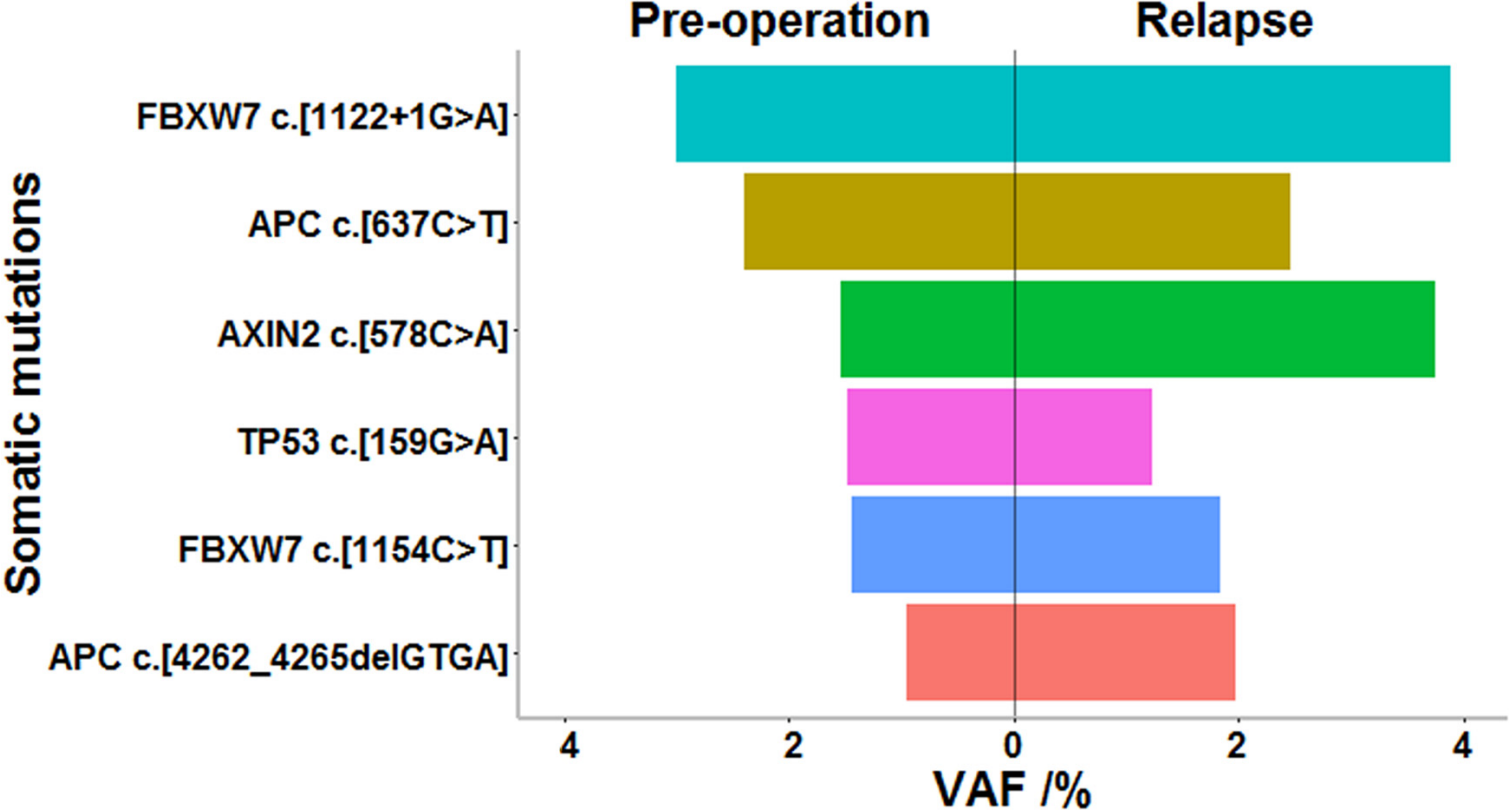
Mutated allele fraction of common somatic variations in preoperative blood versus blood sampled when disease was relapsing of patient 4. VAF, variant allele frequency.

Finally, we compared levels of CEA, CA 19–9, and ctDNA in 4 late-stage patients. CEA and CA 19–9 isolated from pre-operative plasma of patient 3 and patient 4 did not indicate abnormal values (patient 3: CEA = 3.48 μg/L and CA 19–9 = 8.6 kU/L; in patient 4: CEA = 3.72 μg/L and CA 19–9 = 16 kU/L), but ctDNA level was high, along with levels of several mutated driver genes (**Table G in S1 File**). Interestingly, for patient 4, the only patient experiencing relapse, consistent with the results of colonoscopy and CT scan, ctDNA increased 13-fold 18 months post-surgery (**Fig 2**), when levels of CEA and CA 19–9 did not reflect relapse (CEA = 3.8, CA 19–9 = 18.7). Surprisingly, when the tumor was found to recur, mutations in plasma ctDNA were highly consistent with those present in preoperative plasma. Plasma of the relapsing patient harbored all six (100%) somatic mutations seen in the preoperative plasma, of which five were tumor-derived. As shown in **Fig 3**, a higher VAF was found for these mutations, with the exception of TP53.

## Discussion

Similar to previous reports [23, 24], our study described the molecular genetic characteristics of CRC. For example, CRC was associated with mutations in a limited number of driver genes, prominently APC, KRAS, and TP53 [25]. Our results showed that the C>T transition occurred frequently in CRC. This dominant transition was also observed in lung cancer, while not in breast cancer, which may due to the differences in mutagen exposure or DNA-repair processes among cancers[26]. Furthermore, our results suggest that mutations in preoperative plasma and matched tissue differed as a result of spatial intratumor heterogeneity [27]. This heterogeneity could also be responsible for the differences observed between the tumor tissue DNA and the postoperative ctDNA which originated from the residual tumor or metastatic foci.

ctDNA analysis using the next generation sequencing has been demonstrated feasible on the basis of previous researches[11, 28–31]. Some important discoveries have also been reported recently, such as the application of ctDNA analysis for successfully evaluating the early therapeutic response during the treatment of CRC [31] and monitoring the metastatic breast cancer [28]. However, there are still some biological and technological challenges to be conquered for its better application. Firstly, the level of detectable ctDNA fraction varied with tumor type, and that would decrease the sensitivity of this method [14]. Secondly, ctDNA level in peripheral blood changes with disease stage. Patients at higher stages might be identified to carry more variations or an equivalent number of variations while with higher VAF, and these patients would benefit more from this method [32]. This might explain why not all preoperative plasma harbored tumor-derived mutations. On the other hand, the sequencing error rate of ~1% limited the specificity of identifying the low-frequency true mutations [33]. However, with the progression of experimental and analytic methods, errors being introduced during the sample preparation and sequencing have been greatly reduced [34].

Many previous studies reported that one or more mutations in plasma ctDNA changed dynamically along tumor progression [11, 28]. However, different mutations sometimes show different trend of variation while disease progresses, which would confound the accurate interpretation of the data. Newman *et al* introduced a simple new strategy that uses tumor-derived mutations as reporters to evaluate the tumor load of non-small cell lung cancer patients, and their results were consistent with those of CT scan [35]. Taking tumor heterogeneity into consideration, our study described a new strategy of combining tumor-derived and *de novo* mutations in plasma ctDNA to assess the postoperative tumor burden. As the results showed, indicators of recurrence were clearly observed in both ctDNA and imaging data, but not in levels of CEA and CA 19–9, due to the lower sensitivity of the assays for these markers [11, 36, 37]. To our knowledge, this study is the first to report the reappearance of multiple tumor-derived mutations in a relapsing CRC patient, with higher VAF than observed in the plasma ctDNA before surgery. Because tumor progression was accompanied with accumulation of mutations [38], a prominent increase in ctDNA level could be observed earlier than the positive imaging results to indicate disease relapse. Limited in the number of patients and the sensitivity of NGS for detecting mutations in early-stage diseases, we could not analyze the efficacy of this method in this population. However, our study provides evidence that the application of capture sequencing of ctDNA for evaluating the postoperative tumor load should take heterogeneity into consideration, and this may provide a powerful strategy for tracking the progression of both primary and metastatic diseases.

## Conclusions

In this study, we analyzed the molecular genetic characteristics of CRC and described a multi-time-point profile of ctDNA of CRC patients during the course of clinical multimodality treatment. Generally we observed a good correlation of ctDNA level of the patients with their clinical disease status. We therefore proposed the potential of using a new strategy by analyzing the heterogeneous ctDNA to evaluate and monitor the tumor burden of CRC in clinical practice, which may potentially provide better sensitivity and specificity than the traditionally used biomarkers.

## Supporting Information

S1 Fig Somatic nonsynonymous mutations identified in CRC tissue samples. Somatic nonsynonymous mutations (y-axis) identified in tumor tissues of six CRC patients (x-axis) included single-nucleotide variations (SNVs) and small insertions and deletions (indels).
(TIF)

S2 Fig Distribution of somatic SNVs in all tissue samples. (A) Number of SNVs in the indicated transition and transversion categories. (B) Proportion of SNVs in each mutation category.
(TIF)

S3 Fig Sequencing and bioinformatics analysis pipeline of the study. (TIF)

S1 File Supporting tables **Table A in S1 File.** List of target regions **Table B in S1 File.** Clinical characteristics of colorectal carcinoma patients **Table C in S1 File.** Quality control data for tissue and PBL libraries **Table D in S1 File.** Somatic nonsynonymous mutations discovered in tissue by NGS **Table E in S1 File.** Time points of blood sample collection **Table F in S1 File.** Quality control data for plasma libraries **Table G in S1 File.** Somatic nonsynonymous mutations discovered in plasma samples by NGS **Table H in S1 File.** cfDNA and ctDNA in serial plasma samples.
(XLS)

## References

[1] Bohm B, Schwenk W, Hucke HP, Stock W. Does methodic long-term follow-up affect survival after curative resection of colorectal carcinoma? Diseases of the colon and rectum. 1993;36(3):280–6. Epub 1993/03/01. . 8449134 10.1007/BF02053511

[2] Scholefield JH, Steele RJ. Guidelines for follow up after resection of colorectal cancer. Gut. 2002;51 Suppl 5:V3–5. Epub 2002/09/11. 12221030 10.1136/gut.51.suppl_5.v3 PMC1867738

[3] Umpleby HC, Fermor B, Symes MO, Williamson RC. Viability of exfoliated colorectal carcinoma cells. The British journal of surgery. 1984;71(9):659–63. Epub 1984/09/01. . 6478151 10.1002/bjs.1800710902

[4] Bast RC Jr., Ravdin P, Hayes DF, Bates S, Fritsche H Jr., Jessup JM, et al 2000 update of recommendations for the use of tumor markers in breast and colorectal cancer: clinical practice guidelines of the American Society of Clinical Oncology. Journal of clinical oncology: official journal of the American Society of Clinical Oncology. 2001;19(6):1865–78. . 11251019 10.1200/JCO.2001.19.6.1865

[5] Kraus S, Nabiochtchikov I, Shapira S, Arber N. Recent advances in personalized colorectal cancer research. Cancer letters. 2014;347(1):15–21. 10.1016/j.canlet.2014.01.025 . 24491406

[6] Liu Z, Zhang Y, Niu Y, Li K, Liu X, Chen H, et al A systematic review and meta-analysis of diagnostic and prognostic serum biomarkers of colorectal cancer. PloS one. 2014;9(8):e103910 10.1371/journal.pone.0103910 25105762 PMC4126674

[7] Sorbye H, Dahl O. Carcinoembryonic antigen surge in metastatic colorectal cancer patients responding to oxaliplatin combination chemotherapy: implications for tumor marker monitoring and guidelines. Journal of clinical oncology: official journal of the American Society of Clinical Oncology. 2003;21(23):4466–7. 10.1200/JCO.2003.99.200 . 14645446

[8] Diehl F, Li M, Dressman D, He Y, Shen D, Szabo S, et al Detection and quantification of mutations in the plasma of patients with colorectal tumors. Proceedings of the National Academy of Sciences of the United States of America. 2005;102(45):16368–73. Epub 2005/11/01. 10.1073/pnas.0507904102 16258065 PMC1283450

[9] Diehl F, Schmidt K, Choti MA, Romans K, Goodman S, Li M, et al Circulating mutant DNA to assess tumor dynamics. Nature medicine. 2008;14(9):985–90. Epub 2008/08/02. 10.1038/nm.1789 18670422 PMC2820391

[10] Nawroz H, Koch W, Anker P, Stroun M, Sidransky D. Microsatellite alterations in serum DNA of head and neck cancer patients. Nature medicine. 1996;2(9):1035–7. Epub 1996/09/01. . 8782464 10.1038/nm0996-1035

[11] Reinert T, Scholer LV, Thomsen R, Tobiasen H, Vang S, Nordentoft I, et al Analysis of circulating tumour DNA to monitor disease burden following colorectal cancer surgery. Gut. 2015 Epub 2015/02/07. 10.1136/gutjnl-2014-308859 . 25654990

[12] Sherwood JL, Corcoran C, Brown H, Sharpe AD, Musilova M, Kohlmann A. Optimised Pre-Analytical Methods Improve KRAS Mutation Detection in Circulating Tumour DNA (ctDNA) from Patients with Non-Small Cell Lung Cancer (NSCLC). PloS one. 2016;11(2):e0150197 Epub 2016/02/27. 10.1371/journal.pone.0150197 26918901 PMC4769175

[13] Seshagiri S, Stawiski EW, Durinck S, Modrusan Z, Storm EE, Conboy CB, et al Recurrent R-spondin fusions in colon cancer. Nature. 2012;488(7413):660–4. Epub 2012/08/17. 10.1038/nature11282 22895193 PMC3690621

[14] Bettegowda C, Sausen M, Leary RJ, Kinde I, Wang Y, Agrawal N, et al Detection of circulating tumor DNA in early- and late-stage human malignancies. Science translational medicine. 2014;6(224):224ra24 Epub 2014/02/21. 10.1126/scitranslmed.3007094 24553385 PMC4017867

[15] Butler TM, Johnson-Camacho K, Peto M, Wang NJ, Macey TA, Korkola JE, et al Exome Sequencing of Cell-Free DNA from Metastatic Cancer Patients Identifies Clinically Actionable Mutations Distinct from Primary Disease. PloS one. 2015;10(8):e0136407 10.1371/journal.pone.0136407 26317216 PMC4552879

[16] Roschewski M, Dunleavy K, Pittaluga S, Moorhead M, Pepin F, Kong K, et al Circulating tumour DNA and CT monitoring in patients with untreated diffuse large B-cell lymphoma: a correlative biomarker study. The Lancet Oncology. 2015 Epub 2015/04/07. 10.1016/S1470-2045(15)70106-3 . 25842160 PMC4460610

[17] Xu S, Lou F, Wu Y, Sun DQ, Zhang JB, Chen W, et al Circulating tumor DNA identified by targeted sequencing in advanced-stage non-small cell lung cancer patients. Cancer letters. 2016;370(2):324–31. 10.1016/j.canlet.2015.11.005 . 26582655 PMC7495502

[18] Nielsen R, Paul JS, Albrechtsen A, Song YS. Genotype and SNP calling from next-generation sequencing data. Nature reviews Genetics. 2011;12(6):443–51. 10.1038/nrg2986 21587300 PMC3593722

[19] Cibulskis K, Lawrence MS, Carter SL, Sivachenko A, Jaffe D, Sougnez C, et al Sensitive detection of somatic point mutations in impure and heterogeneous cancer samples. Nature biotechnology. 2013;31(3):213–9. 10.1038/nbt.2514 23396013 PMC3833702

[20] Li J, Lupat R, Amarasinghe KC, Thompson ER, Doyle MA, Ryland GL, et al CONTRA: copy number analysis for targeted resequencing. Bioinformatics. 2012;28(10):1307–13. 10.1093/bioinformatics/bts146 22474122 PMC3348560

[21] Chen K, Wallis JW, McLellan MD, Larson DE, Kalicki JM, Pohl CS, et al BreakDancer: an algorithm for high-resolution mapping of genomic structural variation. Nature methods. 2009;6(9):677–81. 10.1038/nmeth.1363 19668202 PMC3661775

[22] Robinson JT, Thorvaldsdottir H, Winckler W, Guttman M, Lander ES, Getz G, et al Integrative genomics viewer. Nature biotechnology. 2011;29(1):24–6. 10.1038/nbt.1754 21221095 PMC3346182

[23] Kandoth C, McLellan MD, Vandin F, Ye K, Niu B, Lu C, et al Mutational landscape and significance across 12 major cancer types. Nature. 2013;502(7471):333–9. 10.1038/nature12634 24132290 PMC3927368

[24] Lawrence MS, Stojanov P, Polak P, Kryukov GV, Cibulskis K, Sivachenko A, et al Mutational heterogeneity in cancer and the search for new cancer-associated genes. Nature. 2013;499(7457):214–8. 10.1038/nature12213 23770567 PMC3919509

[25] Fearon ER. Molecular genetics of colorectal cancer. Annual review of pathology. 2011;6:479–507. 10.1146/annurev-pathol-011110-130235 . 21090969

[26] Wood LD, Parsons DW, Jones S, Lin J, Sjoblom T, Leary RJ, et al The genomic landscapes of human breast and colorectal cancers. Science. 2007;318(5853):1108–13. 10.1126/science.1145720 . 17932254

[27] Sottoriva A, Kang H, Ma Z, Graham TA, Salomon MP, Zhao J, et al A Big Bang model of human colorectal tumor growth. Nature genetics. 2015;47(3):209–16. 10.1038/ng.3214 25665006 PMC4575589

[28] Dawson SJ, Tsui DW, Murtaza M, Biggs H, Rueda OM, Chin SF, et al Analysis of circulating tumor DNA to monitor metastatic breast cancer. The New England journal of medicine. 2013;368(13):1199–209. Epub 2013/03/15. 10.1056/NEJMoa1213261 . 23484797

[29] Klevebring D, Neiman M, Sundling S, Eriksson L, Darai Ramqvist E, Celebioglu F, et al Evaluation of exome sequencing to estimate tumor burden in plasma. PloS one. 2014;9(8):e104417 10.1371/journal.pone.0104417 25133800 PMC4136786

[30] Siravegna G, Mussolin B, Buscarino M, Corti G, Cassingena A, Crisafulli G, et al Clonal evolution and resistance to EGFR blockade in the blood of colorectal cancer patients. Nature medicine. 2015;21(7):827 10.1038/nm0715-827b . 26151329

[31] Tie J, Kinde I, Wang Y, Wong HL, Roebert J, Christie M, et al Circulating Tumor DNA as an Early Marker of Therapeutic Response in Patients with Metastatic Colorectal Cancer. Annals of oncology: official journal of the European Society for Medical Oncology / ESMO. 2015 Epub 2015/04/09. 10.1093/annonc/mdv177 . 25851626 PMC4511218

[32] Lecomte T, Berger A, Zinzindohoue F, Micard S, Landi B, Blons H, et al Detection of free-circulating tumor-associated DNA in plasma of colorectal cancer patients and its association with prognosis. International journal of cancer. 2002;100(5):542–8. 10.1002/ijc.10526 . 12124803

[33] Schmitt MW, Kennedy SR, Salk JJ, Fox EJ, Hiatt JB, Loeb LA. Detection of ultra-rare mutations by next-generation sequencing. Proceedings of the National Academy of Sciences of the United States of America. 2012;109(36):14508–13. 10.1073/pnas.1208715109 22853953 PMC3437896

[34] Kennedy SR, Schmitt MW, Fox EJ, Kohrn BF, Salk JJ, Ahn EH, et al Detecting ultralow-frequency mutations by Duplex Sequencing. Nature protocols. 2014;9(11):2586–606. 10.1038/nprot.2014.170 25299156 PMC4271547

[35] Newman AM, Bratman SV, To J, Wynne JF, Eclov NC, Modlin LA, et al An ultrasensitive method for quantitating circulating tumor DNA with broad patient coverage. Nature medicine. 2014;20(5):548–54. 10.1038/nm.3519 24705333 PMC4016134

[36] Ouyang DL, Chen JJ, Getzenberg RH, Schoen RE. Noninvasive testing for colorectal cancer: a review. The American journal of gastroenterology. 2005;100(6):1393–403. 10.1111/j.1572-0241.2005.41427.x . 15929776

[37] Tao S, Hundt S, Haug U, Brenner H. Sensitivity estimates of blood-based tests for colorectal cancer detection: impact of overrepresentation of advanced stage disease. The American journal of gastroenterology. 2011;106(2):242–53. 10.1038/ajg.2010.393 . 20959816

[38] Bernheim O, Colombel JF, Ullman TA, Laharie D, Beaugerie L, Itzkowitz SH. The management of immunosuppression in patients with inflammatory bowel disease and cancer. Gut. 2013;62(11):1523–8. 10.1136/gutjnl-2013-305300 . 23903238

