# Supplementary figures and images for: Application of Circulating Tumor DNA as a Non-Invasive Tool for Monitoring the Progression of Colorectal Cancer
Source: PLoS One. 2016 Jul 26;11(7):e0159708. doi: 10.1371/journal.pone.0159708 (PMC4961398; doi:10.1371/journal.pone.0159708)

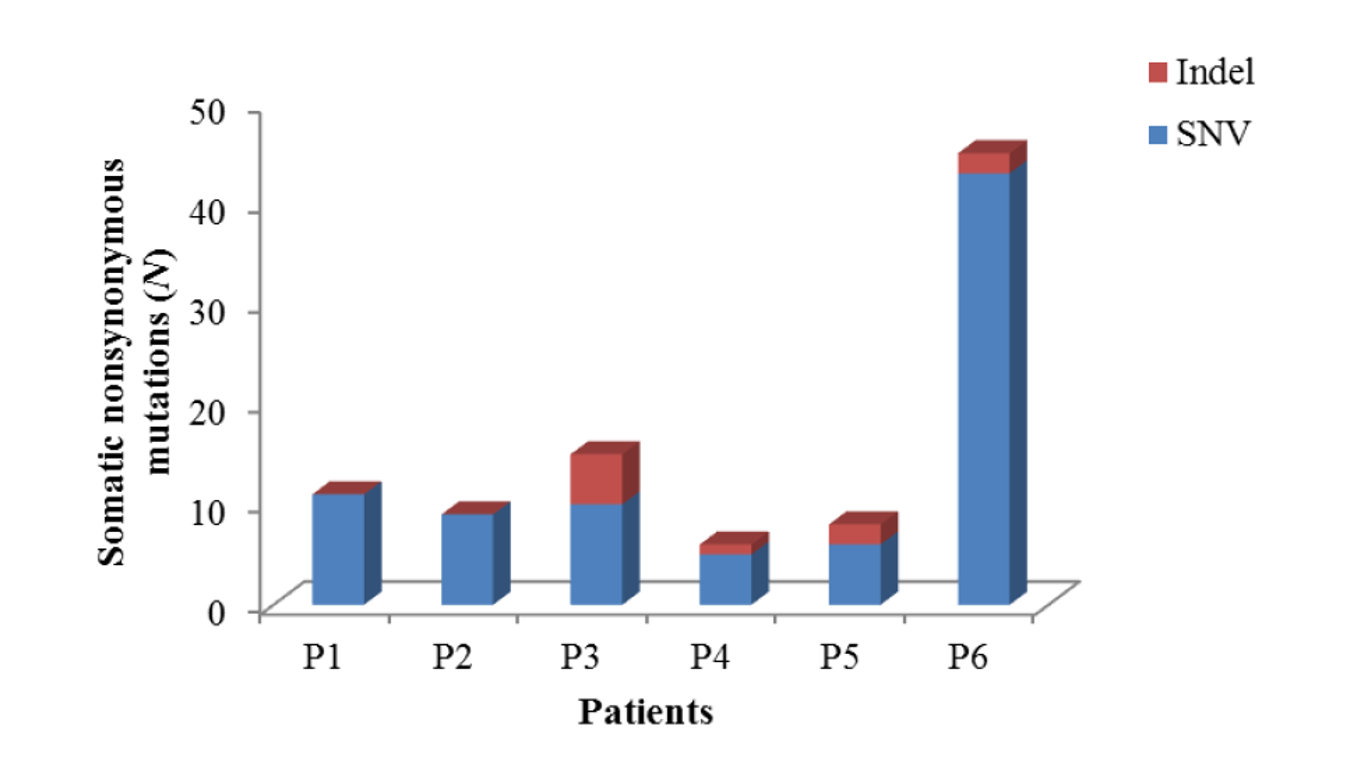

Supplement: S1 Fig — Somatic nonsynonymous mutations (y-axis) identified in tumor tissues of six CRC patients (x-axis) included single-nucleotide variations (SNVs) and small insertions and deletions (indels). (TIF) [file pone.0159708.s001.tif]

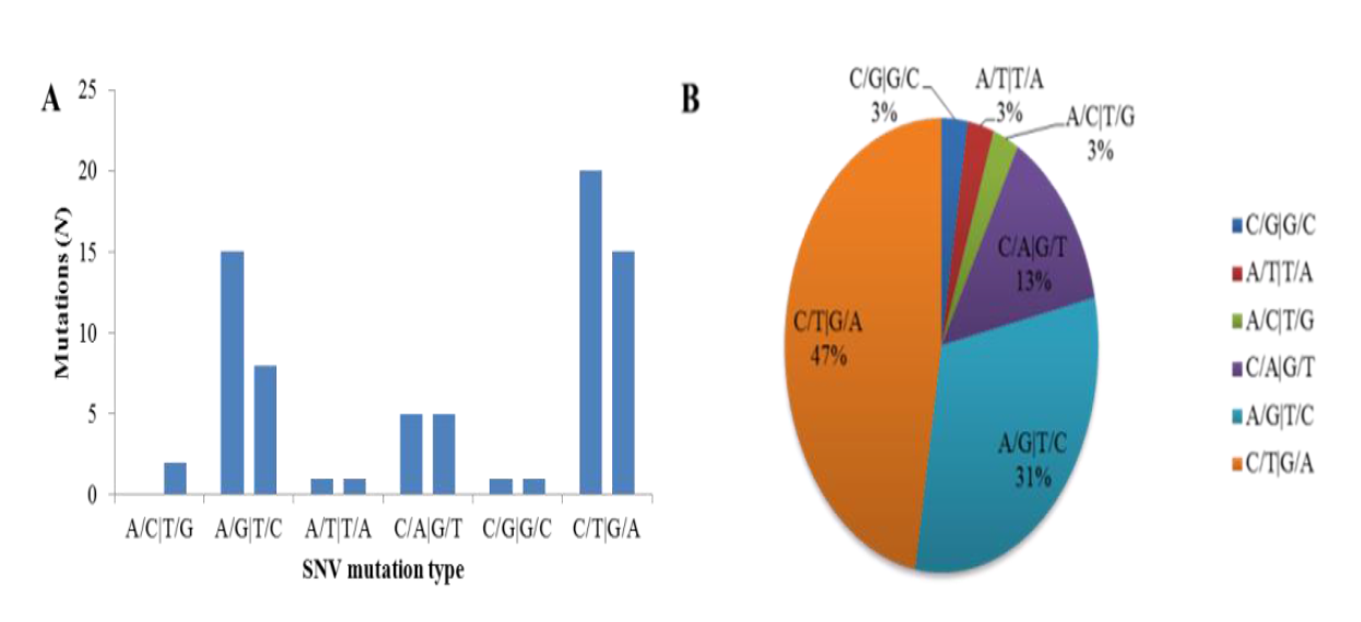

Supplement: S2 Fig — (A) Number of SNVs in the indicated transition and transversion categories. (B) Proportion of SNVs in each mutation category. (TIF) [file pone.0159708.s002.tif]

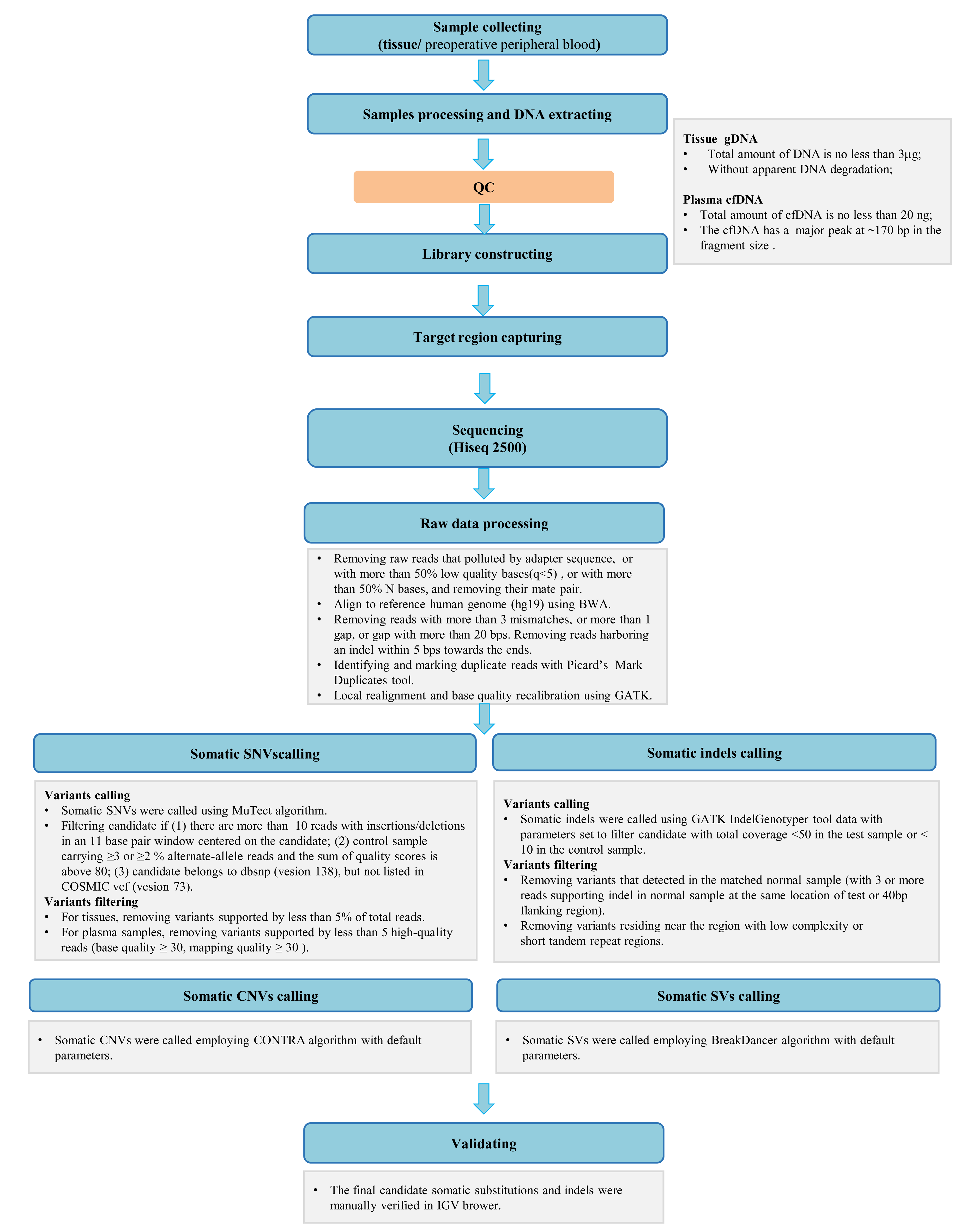

Supplement: S3 Fig — (TIF) [file pone.0159708.s003.tif]
